# Switching among natal and auxiliary hosts increases vulnerability of *Spodoptera exigua* (Hübner) (Lepidoptera: Noctuidae) to insecticides

**DOI:** 10.1002/ece3.2908

**Published:** 2017-03-19

**Authors:** Qamar Saeed, Shafqat Saeed, Faheem Ahmad

**Affiliations:** ^1^ Department of EntomologyFaculty of Agricultural Sciences and TechnologyBZUMultanPakistan; ^2^ Department of EntomologyMNSUAMultanPakistan; ^3^ Department of BiosciencesCOMSATS Institute of Information TechnologyIslamabadPakistan; ^4^ Present address: Faheem Ahmad, State Key Laboratory of Integrated Management of Pest Insects and RodentsInstitute of ZoologyChinese Academy of SciencesBeijing100101China

**Keywords:** beet armyworm management, castor, cauliflower, cotton, host switching, integrated pest management, natal versus auxiliary hosts, okra, polyphagous, spinach

## Abstract

The role of insecticidal application and host plant resistance in managing *Spodoptera exigua* has been well documented, but the effect of different host plants, on which the pest cycles its population in the field, has seldom been investigated. Therefore, we have studied the vulnerability of *S. exigua* against commonly used insecticides (cypermethrin, chlorpyrifos, lufenuron, and emamectin benzoate) with different mode of actions when it switches its generations from natal to auxiliary hosts and vice versa. Different field populations being established on different host plants including castor, cauliflower, cotton, okra, and spinach were collected and reared in the laboratory before insecticidal bioassays. The role of larval diet and host plant switching on their response to tolerate applied insecticides was studied using leaf‐dip bioassay methods. Host switching demonstrated a significant role in altering the vulnerability of *S. exigua* populations to tested insecticides. *Spodoptera exigua* sourced from castor, when switched host to okra and spinach, exhibited 50% higher mortality when treated with emamectin benzoate. This trend in mortality was consistent upon complete host switch cycle (*natal—auxiliary—natal host*). However, the highest increase (92%) in vulnerability was recorded when the larvae were shifted to spinach from cotton. In general, chlorpyrifos and lufenuron had highest efficacies in terms of larval mortality. The findings of present studies provide insights to a better understanding the behavior of polyphagous pests and the role of different host plants in altering the susceptibility of these pests against applied insecticides. Ultimately the results warrant that due consideration should be given to cropping patterns and time of host switching by pest population during planning and executing chemical control.

## 1 Introduction

Among insect pests of cultivated crops, polyphagous insects are most devastating due to the benefit of having alternate refuges to escape from applied management strategies. Beet armyworm, *Spodoptera exigua* Hübner (Lepidoptera: Noctuidae), is one of such insects distributed worldwide (Han, Jin, Kim, & Lee, 2014; Horikiri, 1986; Idris & Emelia, 2001; Sivapragasam & Syed, 2001). Being a polyphagous insect it causes severe economic losses to tomato, cotton, lettuce, celery, cabbage, and alfalfa (Qin, Ye, Huang, Ding, & Lou, 2004). Despite having a range of robust and effective insecticides to be used against *S. exigua* (Ahmad, Arif, & Ahmad, 2007; Antwi & Peterson, 2009), we are unable to control this pest in most of the scenarios. Cosmopolitan distribution of this pest on several cultivated crops and indiscriminate use of broad spectrum insecticides (An, Orellana, Phelan, Cañas, & Grewal, 2016; Armstrong, Abdel‐Mageed, Fokar, Allen, & Adamczyk, 2013) has resulted in the development of insecticide resistance (Sayyed, Ahmad, & Saleem, 2008). The magnitude of economic losses caused by this pest hence warrants the development of, an ecologically acceptable and comprehensive, control and resistance management plan.

Although different strategies have been evaluated for resistance management in the past (Abbas, Shad, & Razaq, 2012; Antwi & Peterson, 2009; Rehan & Freed, 2014), none of them have had a holistic approach, incorporating alternate host plants in the scenario. Understanding of *S. exigua* alternate host use patterns in the field, its ability to switch hosts between generations along the course of its population development, and influences of different host plants on its biological traits could be important in understanding the pests' ecology. Because *S. exigua* is capable of utilizing several wild plants for its population buildup before switching to the primary hosts (Azidah & Sofian‐Azirun, 2006; Berdegué, Reitz, & Trumble, 1998; Futuyma & Moreno, 2003), elucidating the role of alternate hosts on larval and adult performances can lead us to understand the mechanism of resistance development (Panizzi, 1997) and ultimately developing the most effective and rationale chemical management approach against *S. exigua*.

Polyphagous insects have a strong relationship with their host plants from which they get dietary, reproductive, or defensive benefits, selectively using various hosts (Forister, Dyer, Singer, Stireman, & Lill, 2012; Futuyma & Agrawal, 2009; Futuyma & Moreno, 2003). The physio‐chemical characteristics of host plant species affect pests' life cycles either by supplementing their dietary requirements or vice versa (Awmack & Leather, 2002; Simon et al., 2015). Also, host plant has an impact on the body size of the phytophagous insects, ultimately affecting their survival, fecundity, and longevity (Awmack & Leather, 2002).

Commercialization of transgenic cotton varieties (expressing *Bacillus thuringiensis* genes) on one hand has reduced the numbers of sprays against bollworms pests of cotton, but on the other hand, it has induced resurgence in *S. exigua*, which is not affected by the transgenic trait. In the agroecosystems of the northern hemisphere, cotton, being available in field from April to November, supports the initial population of beet armyworm by providing oviposition medium of newly emerged adult females, whereas, in the areas where cotton crop is not available, okra and castor crops are used as auxiliary host by this pest (Ahmad, Ghaffar, & Rafiq, 2013). During autumn, before hibernating, the population shifts on auxiliary hosts including vegetable such as cauliflower and spinach, and also the pest population switches host among these crops when are cultivated in close vicinity. The effect of host switching (utilizing different plant species by progeny of subsequent generations during population buildup and moving on to the primary host during the same cropping season) by polyphagous pest on their susceptibility to chemical control has seldom been explored. As different dietary constituents acquired from different plants influence the physiology of herbivore, we established a hypothesis to test and quantify the role of auxiliary hosts on the efficacy of commonly used insecticides against *S. exigua*.

In this study, we have investigated the efficacy of major insecticides against *S. exigua* which had recently switched auxiliary hosts and have moved to the primary host and vice versa. Our study will help in clearly understanding the role of host plants in resistance management of major phytophagous insect pests and their ecology.

## 2 Materials and Methods

### 2.1 Collection of field populations from different host plants

Five host plants viz. castor (*Ricinus communis* L.), cauliflower (*Brassica oleracea* L.), cotton (*Gossypium hirsutum* L.), okra (*Abelmoschus esculentus* L.*)*, and spinach (*Spinacia oleracea* L.) from cotton growing districts of Pakistan were selected according to host switching regime of *S. exigua* population along the course of its population development. The selected host crops were cultivated in the seasons corresponding to the actual collection of larval population of the pest to ensure the continuous supply of food resources for each population during its laboratory culturing prior bioassays.

To start the master insect cultures for bioassay 500 mixed aged larvae were collected from each selected host. The larvae were reared on the leaves of their natal hosts (hosts from which the initial populations were collected) in the laboratory, and the cultures were maintained at 26 ± 2°C and 60 ± 10% r.h. in 14:10 light: dark regime. The larvae were kept in transparent acrylic jars (10.16 cm in diameter and 20.3 cm high) covered with a piece of muslin cloth. In the laboratory, fresh leaves of their natal hosts were fed to each larva and were replaced every day after sanitizing the jars with ethanol. Pupae were sex‐sorted and paired for oviposition and mass rearing in the laboratory.

Once the master cultures of *S. exigua* were established on each host, we collected the data for relative mortality of larvae of fifth generations in the laboratory, to establish the relative suitability of each host. Ten freshly laid eggs from each population (from each natal host) were kept in a Petri dish (110 mm ø × 20 mm) and were replicated 20 times. Upon larval hatching, daily data for larval mortality on each host were recorded. The larvae from each host were fed with fresh leave of their natal hosts every day.

### 2.2 Bioassays

To study the relationship of the host plant to chemical control of *S. exigua*, chemical insecticides with different modes of action were tested using laboratory bioassays. We selected commonly used conventional insecticides with neurotoxic mode of action, that is, cypermethrin (Arrivo^®^ 10EC, FMC, Pakistan; Na^+^ channel exciters), chlorpyrifos (Lorsban^®^ 40EC; Dow AgroSciences, Pakistan; acetylcholinesterase inhibitor), emamectin benzoate (Proclaim^®^ 1.9EC, Syngenta, Pakistan; Cl^−^ channel activator) and a insect growth regulators (IGRs), lufenuron (Match^®^ 5 EC, Syngenta, Pakistan; chitin biosynthesis inhibitor) for this study. Different concentrations for each insecticide (cypermethrin and chlorpyrifos at 256 ppm, lufenuron at 1.04 ppm and emamectin benzoate at 0.06 ppm) were set based on preliminary studies (Saeed, Saleem, & Ahmad, 2012) and were achieved by diluting with distilled water and nonionic surfactant (Stapple^®^, DuPont, Pakistan) at 5 mg/ml. The control treatment consisted of a mixture of distilled water and surfactant only. As absolutely no mortality of subject larvae was observed 72 hr postapplication in above‐mentioned control treatments (i.e., where no insecticides were applied), the data were excluded from the rest of the analyses. Instead, the data for larval mortality in nonshifting population (no‐shifting: the populations that were not switched to auxiliary hosts) were considered as control (dark gray bars in Figures 2 and 3).

The toxicological bioassays were carried out using leaf‐dip method (Morse, Bellows, & Iwata, 1986). Freshly harvested leaves of all five host plants were cut into disk (4 cm), were dipped into each test concentration and the control solution for at least 10 s, and then allowed to dry in a fume hood for 30 min. Each leaf disk was then placed in Petri with a moistened filter paper at the base to keep the leaf fresh. Five second instar larvae from each population collected from, and reared on, five different hosts were then placed on treated leaf disks of these hosts, and this treatment was replicated eight times (hence a total of 40 larvae per treatment).

The responses of larvae (reared on their natal hosts and diet switched to auxiliary hosts, and then back to their natal hosts) to test insecticides were expressed as a percentage of treated larvae which were dead or moribund at the time of observation. Data for conventional insecticides were recorded 48 hr posttreatment, while for IGRs, it was recorded 72 hr posttreatment.

### 2.3 Host switching among natal and auxiliary hosts

**Figure 1:**
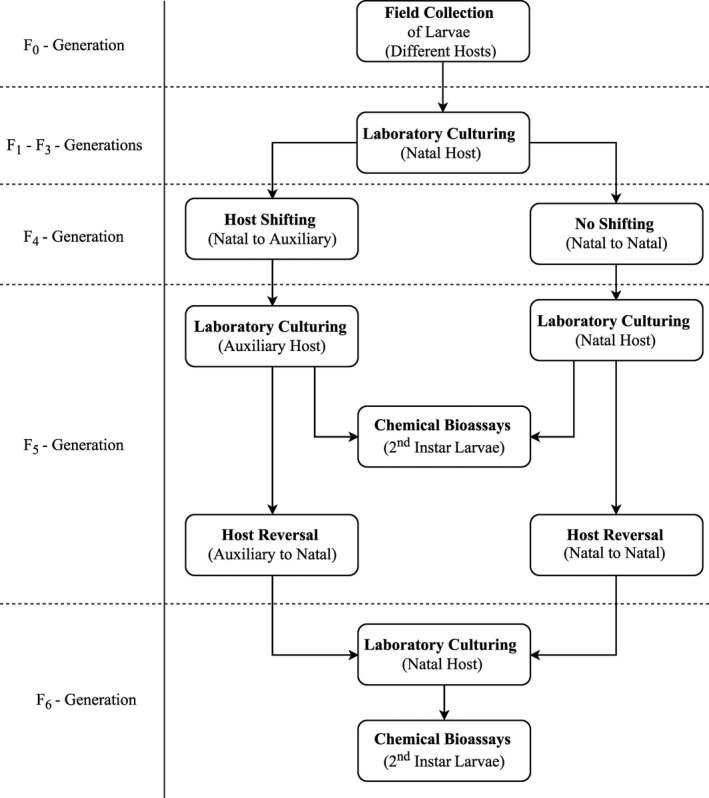
Detailed schema presenting major events in laboratory culturing, host switching and reversal, and bioassays

The above‐mentioned toxicological bioassay was designed to evaluate the role of host shifting during pest life cycle and its effect on the efficiency of chemical control. The complete schema of host shuffling is presented in Figure 1.

The field‐collected populations of *S. exigua* were cultured in the laboratory on their natal host until their fourth generation. Thereafter, F_4_ adults were shifted on to each auxiliary host (each of other four plants selected) for oviposition and population development for one generation (Figure 1). From these F_5_ cultures, second instar larvae were exposed to the insecticides as described above. The data for their mortality on each host treated with different insecticides and a control were recorded.

Once the remaining larvae in F_5_ cultures developed into adults were again moved back to their natal hosts (host reversal) and were cultured for another generation (F_6_). The response of second instar larvae from these cultures was once again tested as described above to account for the effect of host switching between generations along the course of population development in field.

### 2.4 Statistical analyses

The data for relative mortality of *S. exigua* larvae on different hosts in the absence of any chemical treatment were analyzed using a randomized complete block design. Insecticidal bioassays were conducted in completely randomized block—split plot design using host and insecticide treatments as two independent factors and percent mortality of the insects as a dependent factor. The data were subjected to a two‐way ANOVA after testing its conformity to the basic assumption of homogeneity of variances (Levene, 1960), using R‐statistical software (R Development Core Team, 2008). The means were subjected to post hoc pairwise comparisons across hosts and insecticides (*p *≤* *.05; Fisher's LSD with Benjamini–Hochberg correction for multiple pairwise comparisons (Benjamini & Hochberg, 1995).

## 3 Results

### 3.1 Relative suitability of different hosts

No significant difference in egg mortality was observed (*F*_4,99_ **= 1.454; *p *=* *.223); hence, the data are not presented. However, a significant difference in larval mortalities was observed on different hosts, in the absence of any chemical treatment (Table 1). An obvious trend of decrease in mortality was observed from early instars to the later instars (Table 1). Significantly highest numbers of larvae died when reared on okra and cotton, followed by those reared on cauliflower (Table 1). On the other hand, highest numbers of second instar larvae survived when reared on castor. Highest mortality of third instar larvae was observed on castor followed by that on spinach, while all other hosts had shown statistically similar but lower mortality compared to castor and spinach (Table 1). In fourth instar, highest larval mortality was observed in larvae reared on cauliflower while the lowest was in the larvae reared on okra (Table 1). Same trend in larval mortality was observed in fifth instar with an exception, that mortality of larvae on cotton significantly decreased while in the final instar, spinach supported the larvae better than all other hosts (Table 1).

**Table 1:** Percent mortality of *Spodoptera exigua* larvae on different natal hosts in the absence of any insecticidal treatment

	Larval mortality on different hosts					Statistics
	Castor	Cauliflower	Cotton	Okra	Spinach	
1st Instar	11.9 ± 0.67^e^	22.2 ± 0.5^d^	25.8 ± 0.58^c^	29.5 ± 0.72^b^	36.3 ± 0.87^a^	*F*_4,99_ * *= 176.32; *p *<* *.001
2nd Instar	8.5 ± 0.48^d^	16.3 ± 0.99^b^	22.7 ± 0.6^a^	25.3 ± 0.71^a^	13.2 ± 0.34^c^	*F*_4,99_ * *= 1.11.43; *p *<* *.001
3rd Instar	34.2 ± 0.72^a^	14.6 ± 0.94^c^	13.2 ± 0.59^c^	12.1 ± 0.62^c^	19.3 ± 0.51^b^	*F*_4,99_ * *= 172.29; *p *<* *.001
4th Instar	17 ± 0.96^b^	22.9 ± 0.75^a^	16.3 ± 0.51^b^	10.1 ± 0.42^c^	16.6 ± 0.62^b^	*F*_4,99_ * *= 44.84; *p *<* *.001
5th Instar	11.5 ± 0.56^a^	10.1 ± 0.8^a^	4.5 ± 0.2^b^	9.7 ± 0.44^a^	5.8 ± 0.46^b^	*F*_4,99_ * *= 1.45; *p *=* *.001
6th Instar	8.6 ± 1.08^a^	4.5 ± 1.1^ab^	8.2 ± 1.51^a^	5.5 ± 0.88^ab^	3.6 ± 0.96^b^	*F*_4,99_ * *= 3.89; *p *=* *.006

The mean (±*SE*) values in each column followed by different letters in superscript differ significantly from each other (*p* ≤ .05; Fisher's LSD with Benjamini–Hochberg correction for multiple pairwise comparisons).

### 3.2 Host switching from and reversal to castor

**Figure 2:**
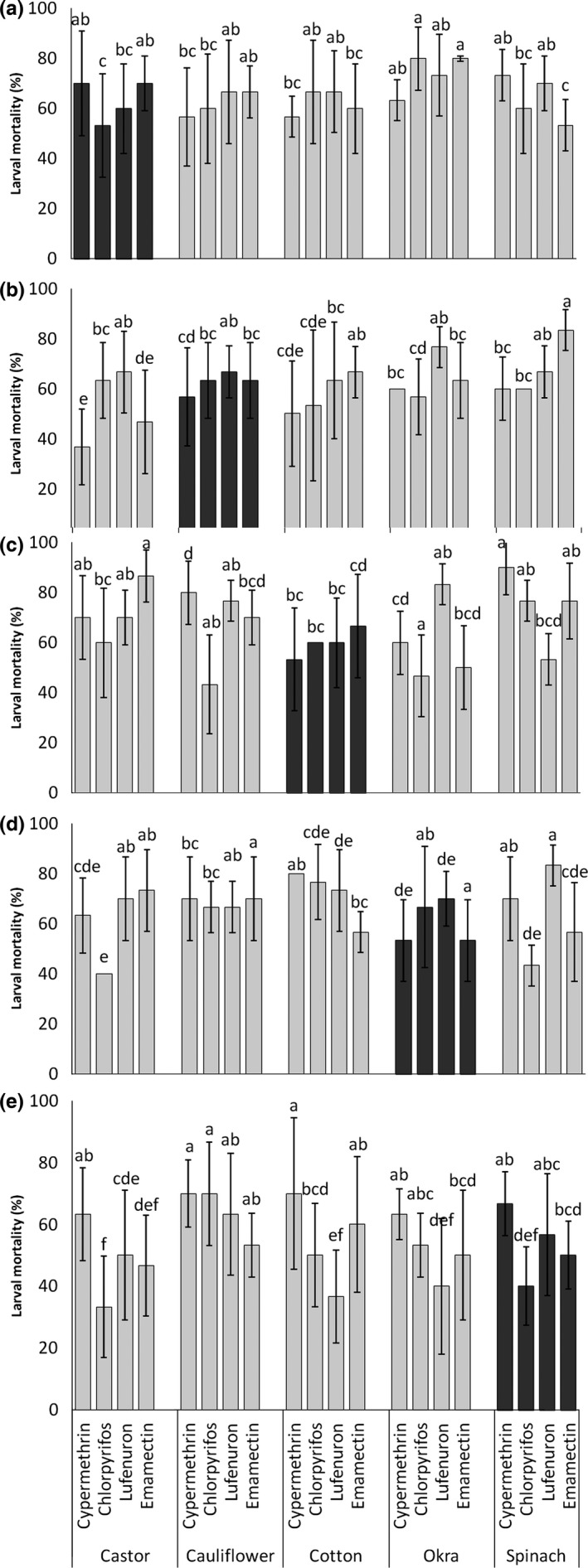
Comparative mortality of *Spodoptera exigua* due to different insecticide treatments on castor (a), cauliflower (b), cotton (c), okra (d), and spinach (e) when no switching from natal host (dark gray bars) and host switching to auxiliary hosts (light gray bars) occurred. Bars represent average percent larval mortality (%), and the error bars are 95% CI. The lowercase letters above bars indicate the outcomes of post hoc pairwise comparisons across hosts and insecticides (*p *<* *.05; Fisher's LSD with Benjamini–Hochberg correction for multiple pairwise comparisons). Bars with the same letter, for each of the comparisons outlined above, were not statistically different from one another as indicated by LSD tests

**Figure 3:**
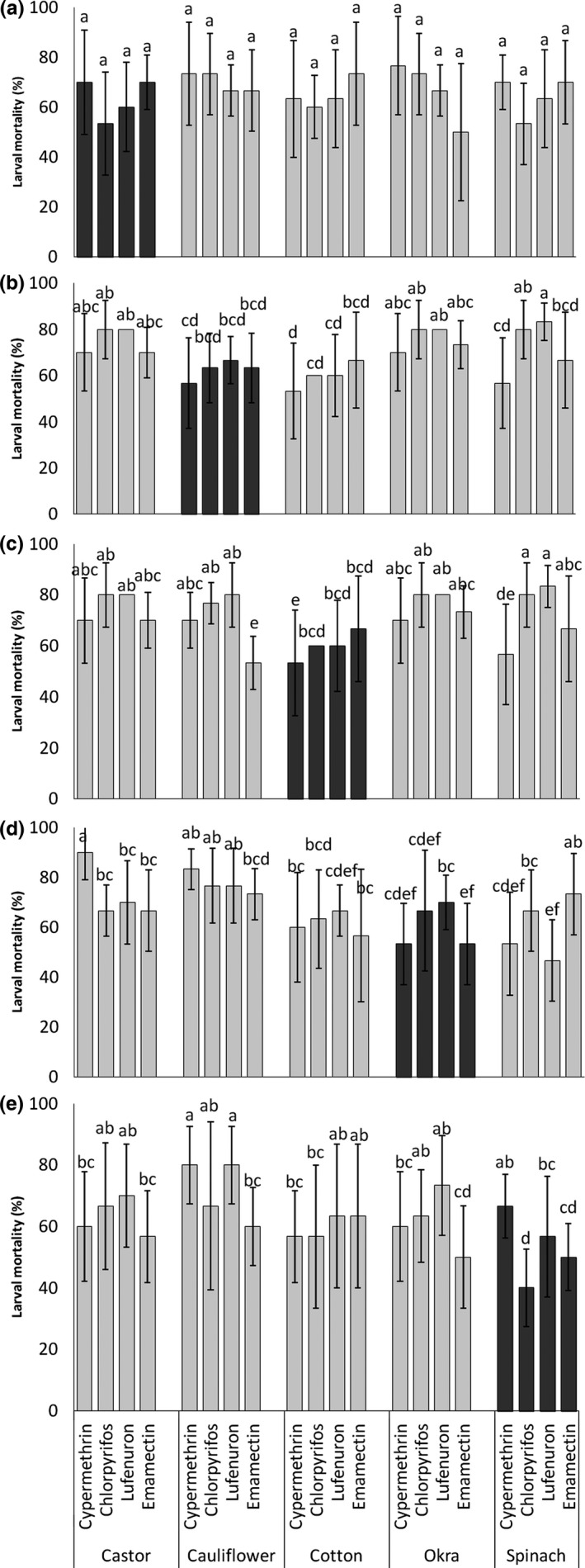
Comparative mortality of *Spodoptera exigua* due to different insecticide treatments on castor (a), cauliflower (b), cotton (c), okra (d), and spinach (e) when no switching from natal host (dark gray bars) and reverse switching from auxiliary hosts to respective natal hosts (light gray bars) occurred. Bars represent average percent larval mortality (%), and the error bars are 95% CI. The lowercase letters above bars indicate the outcomes of post hoc pairwise comparisons across hosts and insecticides (*p *<* *.05; Fisher's LSD with Benjamini–Hochberg correction for multiple pairwise comparisons). Bars with the same letter, for each of the comparisons outlined above, were not statistically different from one another as indicated by LSD tests

The results indicate that when *S. exigua* population switches its host from castor (natal host) to auxiliary hosts (cauliflower, cotton, okra, and spinach), no significant difference in efficacy of insecticides, in terms of percent mortality of the larvae, was observed (*F*_12,100_ **= 1.518, *p* = .13). Only the host species indicated a significant effect on percent mortality of *S. exigua* when treated on auxiliary hosts (*F*_4,100_ **= 2.419, *p* = .05; Figure 2a). The highest mortality of the larvae when treated with insecticides was observed when shifted from castor to okra compared to all other auxiliary hosts (Figure 2a), while on other auxiliary hosts, the mortality was lower than that on okra and nonsignificantly different from each other (Figure 2a). But, the same population, when was switched back to their natal host from auxiliary hosts, the mortality again was not affected by different insecticide treatments (host × insecticide: *F*_12,100_ **= 1.19, *p* = .301; Figure 3a). However, in general, the highest mortality (76.6%) was observed when the larval diet was switched back to castor from okra and treated with cypermethrin (Figure 3a).

**Figure 4:**
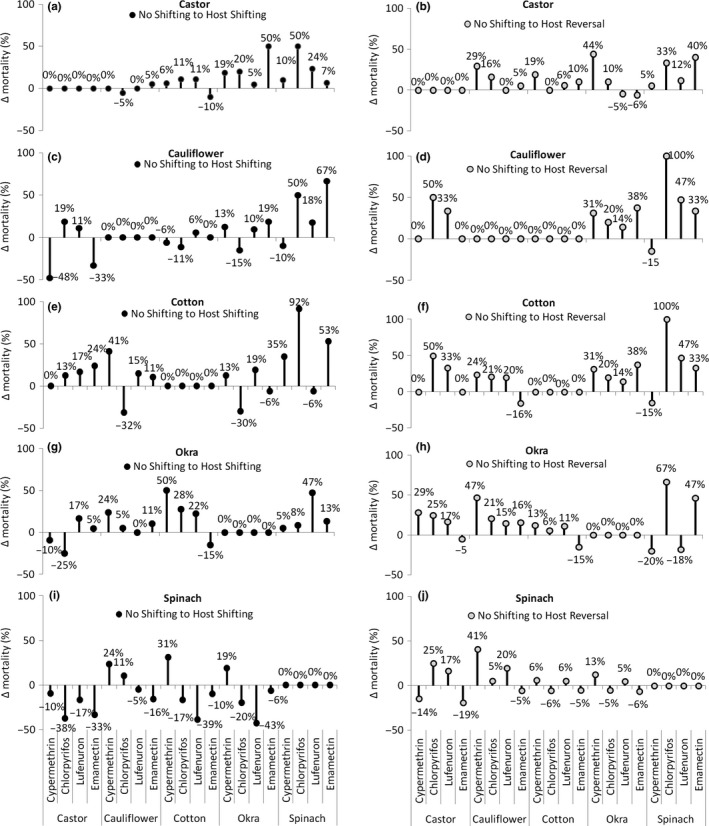
Percent change in mortality of *Spodoptera exigua* due to different insecticidal treatments on various natal hosts due to host shifting (a, c, e, g, i) and after a complete shift cycle (b, d, f, h, j)

The data clearly demonstrate that switching populations from natal to auxiliary hosts increase percent mortality of *S. exigua* larvae, in general (Figure 4a–j). The highest increase in percent mortality (50%) was observed when they were switched from castor to okra and spinach and treated with emamectin benzoate and chlorpyrifos, respectively (Figure 4a).

Percent mortality of the larvae which had a complete host switching cycle (natal host—auxiliary host—natal host) demonstrates an increasing trend in insecticidal efficacy (Figure 4b). Maximum increase in mortality (44%) was observed in the larvae which completed a host shift cycle from castor—okra—castor and was treated with cypermethrin, whereas in the same host shift cycle, when they were treated with lufenuron and emamectin benzoate, the efficacy of insecticides has decreased by 5% and 6%, respectively, compared to those larvae which did not shift hosts (Figure 4b).

### 3.3 Host shifting from and reversal to cauliflower

Percent mortality of the larvae reared on cauliflower (natal host) was significantly influenced by host species (*F*_4,100_ **= 2.92, *p *=* *.023) and insecticides used (*F*_3,100_ **= 5.44, *p *=* *.002) when switched to their auxiliary hosts; however, no significant interaction of host switching and type of insecticide treatment was recorded (*F*_12,100_ **= 1.54, *p *=* *.121; Figure 2b). The highest mortality was observed when these larvae were moved to spinach while the lowest mortality was observed when they were fed on castor (Figure 2b). In terms of insecticidal efficacy, lufenuron caused the highest mortality while the lowest was recorded when they were treated with cypermethrin (Figure 2b).

Upon the reversal of the *S. exigua* population from auxiliary hosts to their natal host (i.e., cauliflower, in this case), both host species and insecticides had a significant effect on mean mortality of the larvae (*F*_4,100_ **= 5.99, *p *<* *.001) and (*F*_3,100_ **= 4.61, *p *=* *.005), respectively. But these both factors did not have show a significant interaction regarding variable larval mortalities (*F*_12,100_ **= 0.66, *p *=* *.786; Figure 3b). Larvae switching back from okra, castor, and spinach to cauliflower (natal host) had statistically similar and highest mortality compared to when they switched back from cotton (Figure 3b). In terms of insecticides, lufenuron and chlorpyrifos show highest but statistically similar mortality to each other (Figure 3b) while cypermethrin had the least efficacy against larvae (Figure 3b).

In terms of the effect of host shifting of the populations from cauliflower to auxiliary hosts in their subsequent generations, the efficacy of cypermethrin decreased in general (Figure 4c). Similarly, the efficacy of chlorpyrifos decreased when the larvae shifted from cauliflower to cotton and okra but increased by 50% when they were shifted to spinach (Figure 4c). A clear increase in mortality due to host shifting was observed when the larvae were shifted to spinach compared to all other auxiliary hosts where a maximum increase in percent mortality has happened when the larvae were treated with emamectin benzoate (Figure 4c). The similar trend was obvious upon the complete switch cycle when the larvae switched back to cauliflower from spinach and the overall mortality increased except where treated with cypermethrin (Figure 4d).

### 3.4 Host switching from and reversal to cotton

Percent mortality of *S. exigua* larvae natal to cotton when switched their population to auxiliary hosts had been significantly affected by the interaction of host species and insecticides (*F*_12,100_ **= 5.289, *p *<* *.001; Figure 2c). The highest and significantly similar mortality was recorded when the larvae switched host to spinach, castor, and okra and treated with cypermethrin, emamectin benzoate, and lufenuron, respectively (Figure 2c). The larvae switched to spinach and okra, in general, had highest mortalities when treated with cypermethrin and lufenuron, respectively (Figure 2c), followed by mortality when switched to castor and okra, and treated with chlorpyrifos and cypermethrin, respectively. The lowest mortality was observed when the larvae from cotton moved to cauliflower and treated with chlorpyrifos, and it was statistically similar to when they were switched to okra and treated with emamectin benzoate and chlorpyrifos (Figure 2c).

Upon host switching back from auxiliary to natal host (cotton, in this case), no significant effect of host species and insecticides interactions was observed (*F*_12,100_ **= 1.38, *p *=* *.186; Figure 3c). Host species and insecticides, however, individually had a significant effect on larval mortality (*F*_3,100_ **= 5.11, *p *<* *.001) and (*F*_4,100_ **= 6.57, *p *=* *.001), respectively. Larval mortality was significantly higher on all auxiliary resources (castor, cauliflower, okra, and spinach) compared to when no switching had happened while lufenuron and chlorpyrifos had a significantly higher mortality of larvae compared to emamectin benzoate and cypermethrin (Figure 3c).

Percent change in larval mortality when they shifted from the natal host (cotton, in this case) demonstrates that a sharp decline in mortality was observed where the larvae were treated with chlorpyrifos on cauliflower (32%) and okra (30%), respectively (Figure 4e). In contrast to that, the same insecticide when applied to the larvae which shifted from cotton to spinach caused a significant increase (92%; Figure 4e). For other insecticides, when applied on the larvae that switched hosts from cotton to castor, cauliflower, okra, and spinach, a generally increasing trend in larval mortality (ranging from 13% to 53%) has been recorded (Figure 4e). Upon analyzing the differences in larval mortality between no host switching larvae (which stayed on cotton) and the ones which complete a switch host cycle (from cotton—auxiliary hosts—cotton), it has been identified that above 33% increase happened when the auxiliary host was castor or spinach (Figure 4f). The maximum increase in larval mortality upon complete shift was observed in larvae returning from spinach (100%) and castor (53%), respectively, when sprayed with chlorpyrifos (100%) while lufenuron cause 33% more larval mortality in both cases (Figure 4f).

### 3.5 Host shifting from and reversal to okra

The larvae when switched hosts from okra to auxiliary resources demonstrated a significant difference in mortality due to the interaction of host species and insecticides (*F*_12,100_ **= 3.77, *p *=* *.002; Figure 2d). In general, average mortality across different insecticide treatments was significant highest when the larvae switched to cauliflower (Figure 2d). Among different insecticide treatments, chlorpyrifos and cypermethrin caused significantly highest mortality when applied on larvae that were switched to cauliflower. This was statistically similar to the mortality observed in larvae switched to cotton when cypermethrin was (Figure 2d). On the other hand, the lowest mortality was observed on cotton and castor when larvae were treated with lufenuron and chlorpyrifos, and it was statistically similar to that in the larvae which did not switch from natal to auxiliary hosts (Figure 2d).

When the insecticides were applied to larvae which switched back from auxiliary hosts to natal host (spinach, in this case), a significant difference in larval mortalities was recorded due to the interaction of host species and insecticides interactions (*F*_12,100_ **= 2.02, *p *=* *.0.03; Figure 3d). The data have demonstrated that highest mean mortality across different host species was observed on cauliflower and castor (Figure 3d) while the lowest mortality was observed in cotton and spinach which were statistically similar to the mortality of the larvae that did not shift their hosts (i.e., stayed on okra; Figure 3d). Larvae of the generation that had returned from cauliflower to its natal host (okra, in this case) had significantly highest mortality compared to other treatments when treated with all four insecticides (Figure 3d). This was statistically similar to mortality caused by chlorpyrifos when applied to larvae returning from castor to okra (Figure 3d). In contrast, the lowest mortality was observed in the larvae returning from spinach when treated with lufenuron and cypermethrin and it was statistically similar to that in nonshifting larvae (Figure 3d).

Percent mortality, in general, increased (5%–50%) when the larvae reared on okra were shifted to cotton and spinach upon application of all insecticides (Figure 4g). Maximum increase was observed when the larvae shifting from okra to cotton and spinach were treated with cypermethrin and lufenuron while percent mortality decreased by 25% and 15% when the larvae switched hosts to castor and cotton and treated with chlorpyrifos and emamectin benzoate, respectively (Figure 4g). Upon completing a host switch cycle, the results demonstrated a reasonable increase in the mortality of larvae which have shifted from cauliflower and spinach back to okra (Figure 4h).

### 3.6 Host shifting from and reversal to spinach

Mortality of the larvae that switched hosts from spinach to other auxiliary hosts (castor, cauliflower, cotton, and okra) was nonsignificantly affected by interaction of host species and insecticides (*F*_12,100_ **= 1.56, *p *=* *.114; Figure 2e); however, it was significantly affected by host species (*F*_4,100_ **= 3.05, *p *=* *.02) and insecticides (*F*_3,100_ **= 7.48, *p *<* *.001), individually. The highest mortality was observed in larvae that switched hosts to cauliflower while all other treatments had statistically similar mortality compared to nonswitching larvae (Figure 2e). Similarly, only cypermethrin demonstrated the best control of larvae compared to rest of the insecticide treatments (Figure 2e).

In terms of host reversal, the mortality of larvae switching back from auxiliary hosts to their natal host (spinach, in this case) no significant effect of host species and insecticide interactions was observed (*F*_12,100_ **= 0.99, *p *=* *.46; Figure 3e). In general, significantly highest mortality was recorded in the larvae shifted from cauliflower, castor, and okra (*F*_4,100_ **= 3.37, *p *=* *.013), while in cotton, it was statistically similar to that in nonswitching larval population (Figure 4e). Similarly, mean mortality across resources due to lufenuron and cypermethrin was significantly higher (*F*_3,100_ **= 3.18, *p *=* *.027) than other two treatments (Figure 3e).

Host switching from spinach (as natal host) to auxiliary hosts has demonstrated a general decreasing trend in insecticidal efficacy in terms of larval mortality (Figure 4i). Only cypermethrin had caused increased mortality of the larvae which were shifted to cauliflower, cotton, and okra (Figure 4i). In contrast, percent mortality of larvae decreased where lufenuron was applied in all cases (Figure 4i). Similarly, a considerable increase in larval mortality was observed only when the larvae completed the switch cycle from castor and cauliflower (back to spinach) and were treated with chlorpyrifos and cypermethrin, respectively (Figure 4j).

## 4 Discussion

*Spodoptera exigua* population switches hosts, along the cropping pattern, between generations, and a change in their vulnerability to applied insecticides occurs. This has been clearly demonstrated in this study, and hence, we strongly suggest that susceptibility of *S. exigua* to insecticides is dependent upon the type of host on which the population has established. In general, percent mortality of the larvae due to insecticide application on castor had been observed to be decreased when the larvae were shifted from cotton (Figure 4e). This could be due to the fact that castor is grown as a perennial crop and *S. exigua* is habitual to consume it as one of the favorable hosts (Ahmad & Iqbal, 2009; Ahmad et al., 2007; Ishtiaq & Saleem, 2011; Rossi, Santos, Carvalho, Alves, & Pereira, 2012). Similarly, when the larvae sourced from all five hosts were switched to spinach, percent mortality due to all insecticides except cypermethrin decreased. However, an increased mortality was attained in the larvae which completed their host‐switch cycle back to their respective natal hosts. These results indicate a clear interaction of chemical composition of the hosts, the larvae feed upon, and insecticidal efficacy. The trend shows that populations that completed a shift cycle involving spinach as an auxiliary host were more susceptible to tested insecticides (Figure 4b,d,f,h) compared to the population on spinach (as natal hosts) when completed a host‐switch cycle from all other auxiliary host species (Figure 4j). The ecdysteroidal components present in spinach may also have a significant role in altering the life cycle of the lepidopteran pest in a way that they may respond to insecticides differently (Sahaf & Moharramipour, 2013). Our results here warrant a detailed further evaluation of the physiological relationship of *S. exigua* to spinach and its role in insecticide resistance.

These findings indicate that *S. exigua* can refuge easily in spinach. We may suggest that the best time to control this pest would be after spinach is harvested from the fields and the pest population has moved back to their natal host or another auxiliary host. But it all depends on the physical characteristics of the varieties, such as trichomes and glossy surfaces which certainly do not favor the lepidopteron pests (Adamski, Niewadzi, & Ziemnicki, 2005; Adamski et al., 2009; Ahmad et al., 2007; Álvarez, Pera, Loto, Virla, & Baigori, 2009; Álvarez‐Alfageme, Ortego, & Castañera, 2009; Antwi & Peterson, 2009; Atwal & Dhaliwal, 2013; Javed, Aziz, & Leghari, 2009).

On reversal sets of cotton, the larvae returning from castor, spinach, cauliflower, and okra showed, in general, that the control of larvae by treating with cypermethrin and emamectin has decreased. These facts might be attributed to the indiscriminate use of insecticides in cotton crop that is why the cotton‐sourced larvae when shifted to auxiliary hosts show more resistance against insecticides tested. Also, increase in transgenic cotton cultivated area in Pakistan can be considered the main culprit in inducing resistance in the pest (Yang, Li, & Wu, 2013) due to exposure of *S. exigua* to the sublethal dosage of insecticides applied against sucking insect pests of cotton.

The overall scenario of insecticide efficacy against the cauliflower‐sourced larvae depicted that there was a minimum change in mortality when those larvae shifted their hosts to castor, cotton, and okra, except when they were shifted to spinach. Plants from Brassicaceae are considered to be susceptible hosts to *S. exigua*, and the female moths prefer these crops for oviposition (Layton, 1994; Sarfraz, Dosdall, & Keddie, 2007; Van Laecke & Degheele, 1991). This may be the case here in our studies leading to high resistance against insecticides in cauliflower‐sourced larvae.

From these findings, we have clearly demonstrated that the response of *S. exigua* changes as they move along different host plants in the field. Therefore, it can be concluded that cropping pattern should be given due importance in selecting the insecticide and time of insecticide application. Chlorpyrifos and emamectin benzoate have demonstrated increased efficacy, expressed in terms of percent mortality of treated larvae, whenever the host shift cycle involved spinach as auxiliary host. On contrary, the efficacy of these insecticides is not affected significantly when the larvae start their lifecycle from spinach and are shifted to other hosts. These results warrant an exploration of nutritive contents of spinach as a food resource for *S. exigua*. The presence of sterols and other metabolites in spinach has already been reported to cause a behavioral change in herbivore insects by interfering with their hormone systems (Rosenthal & Berenbaum, 1992). The life cycles of the insects feeding on plants are directly proportionate to the characters of host plants on which they are feeding (Awmack & Leather, 2002). The variations showed in our results, in terms of variable mortality of larvae, could be the indications of either difference of qualitative nutrition or quantitative nutrition of host plants species which were used to feed the *S. exigua* (Bernays & Chapman, 1994). At the same time, the variation in environmental conditions during the experiment, particularly fluctuation of temperature and ages of plants used, cannot be neglected while drawing conclusions (Wada, 1979). These changes usually cause fluctuations in life history traits of the herbivore by prolonging stadia, increasing number of molts or vice versa (Azidah & Sofian‐Azirun, 2006).

The results of this study provide a new perspective to integrated management of beet armyworm. Here, we suggest that the knowledge of local cropping patterns and time of host switching by pest population could be accounted for during planning the management strategies. This further warrants the understanding of the physiological mechanism underlying the phenomenon of increased vulnerability of the pest to insecticides after host switching.

## Conflict of Interest

None declared.

## Authors' Contributions

Q.S. and S.S. designed the study; Q.S. and F.A. collected field specimens and managed laboratory mass culturing; Q.S. performed biological and toxicological experiments; F.A. performed data analysis and interpretation; Q.S. and F.A. wrote the manuscript.

## Data Accessibility

Data available from the Dryad Digital Repository entitling, “Data from: Switching among natal and auxiliary hosts increases vulnerability of Spodoptera exigua (Hübner) (Lepidoptera: Noctuidae) to insecticides”. doi:10.5061/dryad.2fn1k.
